# Genetic susceptibility of opioid receptor genes polymorphism to drug addiction: A candidate-gene association study

**DOI:** 10.1186/s12888-020-03006-z

**Published:** 2021-01-05

**Authors:** Laith N. AL-Eitan, Doaa M. Rababa’h, Mansour A. Alghamdi

**Affiliations:** 1 Department of Applied Biological Sciences, Jordan University of Science and Technology, Irbid, 22110 Jordan; 2 Department of Biotechnology and Genetic Engineering, Jordan University of Science and Technology, Irbid, 22110 Jordan; 3 Department of Anatomy, College of Medicine, King Khalid University, Abha, 61421 Saudi Arabia; 4 Genomics and Personalized Medicine Unit, College of Medicine, King Khalid University, Abha, 61421 Saudi Arabia

**Keywords:** Opioids, Polymorphism, Drug addiction, Jordan

## Abstract

**Background:**

Like other complex diseases including drug addiction, genetic factors can interfere with the disease. In this study, three opioid genes (*OPRM1*, *OPRD1,* and *OPRK1*) were examined for an association with drug addiction among Jordanian males.

**Methods:**

The study involved 498 addicts, in addition to 496 healthy controls and all from Arab descent.

**Results:**

The findings in this study showed that rs1799971 of the *OPRM1* gene was in association with drug addiction for both alleles and genotypes with *P*-values = 0.002 and 0.01, respectively. In addition, a significant association between the dominant model (A/A vs G/A-G/G) of rs1799971 (*OPRM1*) and drug addiction (*P*-value = 0.003, OR = 1.59 (1.17–2.15)) was detected. Moreover, a genetic haplotype (AGGGCGACCCC) of the*OPRM1* gene revealed a significant association with drug addiction (*P*-value = 0.01, OR = 1.56 (1.15–2.12)). We also found that the age of addicts, smoking, and marital status with genetic variants within *OPRM1*, *OPRD1,* and *OPRK1* genes may be implicated in drug addiction risk.

**Conclusion:**

We propose that rs1799971 of the *OPRM1*gene is a genetic risk factor for drug addiction among Jordanian males.

**Supplementary Information:**

The online version contains supplementary material available at 10.1186/s12888-020-03006-z.

## Background

Drug addictions are chronic complex diseases that featured by repetitive and irresistible consumption of certain drugs that lead to temporary euphoria. Subsequently, the addict will encounter tolerance, craving, and relapse as a result of neurological changes [1]. Up to 5% of the adults worldwide have been reported as drug abusers in (2010), drug addiction burden both society and individual’s life in many aspects including physical, mental, and social aspects. Moreover, over 200 thousand deaths annually are recording due to drug addiction [2]. Addiction has been linked to environmental factors together with the genetic one [3]. Inspecting the role of genetic variations in the etiology of the addiction may improve response to the treatments and help in disease prevention [4, 5]. Both genome-wide methods and candidate gene studies, looking for the underlying role of the genetic factor in drug addiction by identification of genes that are crucial to neuroadaptation [6].

Opioid receptors (OPR) are part of the opioid system and responsible for mediating drug reward and addiction. These receptors in the nervous system attached to substances and induce a series of chemical reactions that lead to pain relief [7]. The *OPRM1* gene located on chromosome number 6 and encode for endogenous G protein-coupled mu-opioid receptors [8, 9]. The *OPRM1* is one of the most studied genes regarding drug addiction and several genetic variants within the gene were significantly implicated with drug addiction. (rs1799971) SNP of the *OPRM1* gene has been extensively investigated in association with drug addiction in different ethnic groups including Caucasian [10] European American, African American [11], German [12], and Japanese [13].

In addition, the *OPRD1* and *OPRK1* genes encode for the δ-opioid receptor (DOR) and κ-opioid receptor (KOR) respectively are also belong to the opioid system. The *OPRD1*gene located on chromosome 1, while the *OPRK1* gene located on chromosome 8 [14]. Activation of the μ-opioid receptor and δ-opioid receptor mediate drug rewarding DOR has been assigned as mood regulator for anxiety and depression [15]. Within the *OPRD1* gene, both rs1042114 (G80T) and rs2234918 (T921C) have been in the spotlight for association with drug addiction. The latter SNP found to be correlated with heroin addiction in a German population [16]. Furthermore, the two intronic SNPs (rs2236857 and rs581111) within the *OPRD1* gene found to be linked with heroin addiction risk [17].

Areas κ-opioid receptor activation is associated with increases in both physical and psychological stress responses. Genetic polymorphisms of the *OPRK1* gene have been previously studied in association with substances [18]. In this study, we investigated the genetic susceptibility of three OPR genes (*OPRM1*, *OPRD1* and *OPRK1*) to drug addiction among Jordanian males by screening and analyzing 29 candidate SNPs.

## Methods

### Participants

The study cohort consisted of 498 drug addicts selected depending on drug addiction criteria according to the Manual of Mental Disorders (DSM-IV) criteria [19]. All participants were males from Jordanian and were hospitalized in 2018 for 8 months in the National Centre for Rehabilitation of Addicts (NCRA) of the ministry of health in Jordanian and the Drug Rehabilitation Centre of the Jordanian Public Security Directorate (DRC-PSD). In addition, 496 healthy Jordanian male subjects with no history of drug addiction or psychiatric disorders were chosen as controls. Clinical data and structured questionnaires were collected according to the Human Ethics Committee of the Jordanian Ministry of Health (MOH/REC/180057), and the Institutional Review Board (IRB) at Jordan University of Science and Technology (43/114/2018). Patients who meet the inclusion criteria and had none of the exclusion criteria were interviewed, had the study objectives explained to them, and were asked to sign a consent form to participate in the study. Required written informed consent has been also obtained from all participants. All methods of the current study were performed in accordance with the IRB guidelines and regulations. Finally, all data was coded and no specific individual was identified.

### SNPs selection

In this study, the 29 SNPs of three OPR genes (*OPRM1*, *OPRD1*, and *OPRK1*) were selected based on their clinical significance and according to early publications that reveled the plausible implication of these variants with drug addiction among other populations which make them polymorphisms of interest to be investigated as most of them have robust functions in neuroadaptation [20, 21]. Moreover, these polymorphisms selected to declare their influence on the Jordanian population of Arab descent.

### DNA extraction and SNP genotyping

Venous blood samples were used to purify genomic DNA according to Wizard® Genomic DNA Purification Kit (Promega Corporation, USA). Agarose gel electrophoresis and the Nano-Drop ND-1000 UV-Vis Spectrophotometer (BioDrop, UK) were used to select the quality and quantity of the extracted genetic material. Candidate SNPs within (*OPRM1, OPRD1,* and *OPRK1*) were screened using the Sequencing technique. Selected samples for genotyping were diluted using nuclease-free water with a final concentration of 20 ng/μl (50-500 μl) and shipped on wet ice to the Australian Genome Research Facility (AGRF) (Australia). At the AGRF, the samples were genotyped using the Agena Bioscience MassARRAY® on a Compact Spectrometer, iPLEX GOLD chemistry.

### Statistical analysis

The *P*-Value of Hardy-Weinberg equilibrium (HWE), was used to detect if the selected variants fulfill the (HWE) equation. Genotypic and allelic frequencies, genetic association, multiple genetic models, and haplotyping analyses were performed using SNPStats software (InstitutCatalàd’Oncologia, 2006). In addition, multinomial logistic regression was done using the Statistical Package for the Social Sciences (SPSS), version 25.0 (SPSS, Inc., Chicago, IL). *P*-values less than 0.05 were considered to be statistically significant.

### Multiple testing corrections

The effective number of SNPs were tested according to [22] method, in addition, the Bonferroni correction was used to sets the significance cut-off at α/n where α = 0.05 and n number of tests [23].

## Results

### Clinical characteristic of participants

All participants in this study were Jordanian males of Arab descent. Four hundred ninety-eighr cases were identified as drug addicts with respect to different substances including; synthetic cannabinoids (47.5%), cannabinoids (19.6%), amphetamine (5.7%), alcohol (5.5%), benzodiazepines (4.6%), opiates (4.4%), cocaine (1.1%), and cannabis (0.4%). Among the cases 89% used only one substance. However, 11% of the addicts in this study were multi substance user. The routes for substances consumption were varying between substances. For instance, smoking was commonly used for cannabinoids use, while opiate was administrated by the cases using injection route. In addition, benzodiazepines, alcohol, and amphetamine were consumed orally. Cases were using inhalation for cocaine substance use.

The range of study cohort’s ages was from 18 to 40 years with mean age 29 ± 6.9 for controls and 28.7 ± 9.3 for patients. Age, smoking, work, and marital status for cases were considered also in this study; 88.2% of the patients were smokers, (70.4%) were single and (26.8%) were unemployed.

### SNPs characterization

Table 1 summarizes the list of selected genetic variants of three OPR genes (*OPRM1*, *OPRD1*, and *OPRK1*). The table also shows the chromosomal positions of SNPs, minor alleles and their frequencies, and the *P*-value of HWE for cases and controls. Among the investigated SNPs, rs495491 SNP of the *OPRM1* gene did not fulfill the HWE eq. (*P*-value < 0.05) and were excluded from this study.

**Table 1:** SNPs information and minor allele frequencies among cases and controls

Gene	SNP ID	SNP position^**a**^	Cases (***n*** = 498)			Controls (***n*** = 496)		
			MA^**b**^	MAF^**c**^	HWE^**d**^ ***P***-value	MA^**b**^	MAF^**c**^	HWE^**d**^ ***P***-value
*OPRM1*	rs648893	6:154117494	G	0.26	0.29	G	0.27	0.49
	rs609148	6:154109880	A	0.26	0.56	A	0.28	0.91
	rs495491	6:154061407	G	0.21	0.02	G	0.21	0.14
	rs3823010	6:154058017	A	0.08	1.00	A	0.09	0.58
	rs1799971	6:154039662	G	0.14	1.00	G	0.10	0.80
	rs511435	6:154047412	T	0.16	0.5	T	0.18	0.36
	rs524731	6:154053957	A	0.15	0.39	A	0.17	0.63
	rs1381376	6:154072123	T	0.08	1.00	T	0.1	0.30
	rs3778156	6:154083178	G	0.08	0.54	G	0.09	0.59
	rs2075572	6:154090869	G	0.44	0.27	G	0.48	0.24
	rs548646	6:154097012	T	0.32	0.41	T	0.34	0.32
	rs671531	6:154119607	A	0.32	0.68	A	0.34	0.16
*OPRK1*	rs12675595	8:53255365	A	0.1	0.79	A	0.1	0.44
	rs1051660	8:53251002	A	0.11	0.64	A	0.12	0.53
	rs6985606	8:53248556	T	0.33	1.00	T	0.33	1.00
	rs997917	8:53239818	C	0.46	0.59	C	0.44	0.14
	rs702764	8:53229597	C	0.20	0.89	C	0.20	0.21
	rs963549	8:53229264	T	0.2	1.00	T	0.2	0.21
*OPRD1*	rs569356	1:28810174	G	0.08	0.75	G	0.07	1.00
	rs1042114	1:28812463	G	0.08	1.00	G	0.08	0.19
	rs678849	1:28818676	T	0.46	0.36	T	0.45	0.86
	rs2236857	1:28835097	C	0.35	0.14	C	0.38	1.00
	rs2236855	1:28835487	A	0.34	0.13	A	0.38	1.00
	rs2298896	1:28839626	G	0.41	0.30	G	0.45	0.59
	rs421300	1:28843081	G	0.42	0.58	G	0.45	0.32
	rs529520	1:28848434	C	0.46	0.27	C	0.43	0.07
	rs12749204	1:28849701	G	0.23	0.61	G	0.25	0.72
	rs2234918	1:28863085	T	0.46	0.86	T	0.46	0.79

^a^ Chromosomal positions. ^b^ *MA* minor allele. ^c^ *MAF* minor allele frequency, ^d^ *HWE* Hardy—Weinberg equilibrium

### Genetic association analyses

The correlation between the studied SNPs and drug addiction was conducted by performing different statistical tests. Table 2 illustrates the differences in the allelic and genotypic distribution between patients and controls. Our findings exposed that only rs1799971 of the *OPRM1* gene was in association with drug addiction in this study (*P*-values were; 0.002, 0.01) for alleles and genotypes difference respectively. A significant variation in the variant allele (G) frequency distribution was observed between cases and controls; 14% of patients carried the (G) allele compared to only1% of healthy participants with the same variant (Table 2). In addition, the genotype (GG) among patients was twice it among control, this finding may propose the (G) allele of rs1799971 (*OPRM1*) as a risk genetic locus for drug addiction. Moreover, different genetic models analysis was done for further confirmation. Table S**1** displays the included models in this study (dominant and recessive). According to Bonferroni correction, the *P*-value was set to 0.02. The result show a significant association between the dominant model (A/A vs G/A-G/G) of rs1799971 (*OPRM1*) and drug addiction (*P*-value = 0.003, OR = 1.59 (1.17–2.15)). Haplotyping was also investigated as a part of the genetic association’s analyses in this study. As Table S2 demonstrates, one block of the *OPRM1* gene (AGGGCGACCCC) exhibits significant association with drug addiction (*P*-value = 0.01, OR = 1.56 (1.15–2.12)).

**Table 2:** Association of the investigated candidate gene polymorphisms with drug addiction

Gene	SNP ID	Allelic and Genotypic Frequencies in Cases and Controls				
		Allele/Genotype	Cases (***n*** = 498)	Controls (***n*** = 496)	***P***-value*	Chi-square
*OPRM1*	rs648893	A	734 (0.74)	723 (0.73)	0.57	0.31
		G	256 (0.26)	267 (0.27)		
		AA	267 (0.54)	267 (0.54)	0.34	2.1
		AG	200 (0.4)	189 (0.38)		
		GG	28 (0.06)	39 (0.08)		
	rs609148	G	729 (0.74)	709 (0.72)	0.24	1.3
		A	255 (0.26)	279 (0.28)		
		GG	267 (0.54)	255 (0.52)	0.42	1.7
		GA	195 (0.4)	199 (0.4)		
		AA	30 (0.06)	40 (0.08)		
	rs3823010	G	905 (0.92)	901 (0.91)	0.44	0.58
		A	79 (0.08)	89 (0.09)		
		GG	416 (0.85)	411 (0.83)	0.68	0.75
		GA	73 (0.15)	79 (0.16)		
		AA	3 (0.01)	5 (0.01)		
	rs1799971	A	848 (0.86)	894 (0.9)	0.002	9.4
		G	140 (0.14)	96 (0.1)		
		AA	364 (0.74)	404 (0.82)	0.01	9.3
		GA	120 (0.24)	86 (0.17)		
		GG	10 (0.02)	5 (0.01)		
	rs511435	C	833 (0.84)	811 (0.82)	0.18	1.7
		T	155 (0.16)	177 (0.18)		
		CC	353 (0.71)	336 (0.68)	0.42	1.7
		CT	127 (0.26)	139 (0.28)		
		TT	14 (0.03)	19 (0.04)		
	rs524731	C	835 (0.85)	824 (0.83)	0.43	0.60
		A	151 (0.15)	164 (0.17)		
		CC	356 (0.72)	345 (0.7)	0.71	0.67
		CA	123 (0.25)	134 (0.27)		
		AA	14 (0.03)	15 (0.03)		
	rs1381376	C	907 (0.92)	893 (0.9)	0.12	2.4
		T	77 (0.08)	97 (0.1)		
		CC	418 (0.85)	405 (0.82)	0.25	2.7
		CT	71 (0.14)	83 (0.17)		
		TT	3 (0.01)	7 (0.01)		
	rs3778156	A	909 (0.92)	899 (0.91)	0.34	0.9
		G	79 (0.08)	91 (0.09)		
		AA	419 (0.85)	409 (0.83)	0.64	0.88
		AG	71 (0.14)	81 (0.16)		
		GG	4 (0.01)	5 (0.01)		
	rs2075572	C	550 (0.56)	518 (0.52)	0.11	2.5
		G	434 (0.44)	472 (0.48)		
		CC	160 (0.33)	142 (0.29)	0.30	2.4
		CG	230 (0.47)	234 (0.47)		
		GG	102 (0.21)	119 (0.24)		
	rs548646	C	648 (0.68)	640 (0.66)	0.33	0.93
		T	310 (0.32)	336 (0.34)		
		CC	223 (0.47)	215 (0.44)	0.63	0.91
		CT	202 (0.42)	210 (0.43)		
		TT	54 (0.11)	63 (0.13)		
	rs671531	G	669 (0.68)	653 (0.66)	0.33	0.92
		A	313 (0.32)	335 (0.34)		
		GG	230 (0.47)	223 (0.45)	0.51	1.35
		GA	209 (0.43)	207 (0.42)		
		AA	52 (0.11)	64 (0.13)		
*OPRK1*	rs12675595	G	897 (0.9)	894 (0.9)	0.93	0.01
		A	95 (0.1)	96 (0.1)		
		GG	406 (0.82)	405 (0.82)	0.95	0.1
		GA	85 (0.17)	84 (0.17)		
		AA	5 (0.01)	6 (0.01)		
	rs1051660	C	882 (0.897)	869 (0.88)	0.36	0.83
		A	108 (0.11)	121 (0.12)		
		CC	383 (0.77)	394 (0.8)	0.66	0.81
		CA	103 (0.21)	94 (0.19)		
		AA	9 (0.02)	7 (0.01)		
	rs6985606	C	217 (0.67)	664 (0.67)	NA	NA
		T	327 (0.33)	324 (0.33)		
		CC	217 (0.44)	223 (0.45)	0.96	0.07
		CT	219 (0.45)	218 (0.44)		
		TT	54 (0.11)	53 (0.11)		
	rs997917	T	529 (0.54)	552 (0.56)	0.30	1.08
		C	455 (0.46)	432 (0.44)		
		TT	139 (0.28)	163 (0.33)	0.20	3.2
		TC	251 (0.51)	226 (0.46)		
		CC	102 (0.21)	103 (0.21)		
	rs702764	T	788 (0.8)	790 (0.8)	0.91	0.01
		C	202 (0.2)	200 (0.2)		
		TT	314 (0.63)	320 (0.65)	0.69	0.72
		TC	160 (0.32)	150 (0.3)		
		CC	21 (0.04)	25 (0.05)		
	rs963549	C	789 (0.8)	783 (0.8)	0.73	0.11
		T	195 (0.2)	201 (0.2)		
		CC	316 (0.64)	316 (0.64)	0.62	0.93
		CT	157 (0.32)	151 (0.31)		
		TT	19 (0.04)	25 (0.05)		
*OPRD1*	rs569356	A	910 (0.92)	912 (0.93)	0.73	0.11
		G	74 (0.08)	70 (0.07)		
		AA	421 (0.86)	423 (0.86)	0.89	0.23
		AG	68 (0.14)	66 (0.13)		
		GG	3 (0.01)	2 (0.01)		
	rs1042114	T	912 (0.92)	912 (0.92)	NA	NA
		G	76 (0.08)	76 (0.08)		
		TT	421 (0.85)	423 (0.86)	0.73	0.62
		TG	70 (0.14)	66 (0.13)		
		GG	3 (0.01)	5 (0.01)		
	rs678849	C	527 (0.54)	545 (0.55)	0.47	0.51
		T	455 (0.46)	441 (0.45)		
		CC	136 (0.28)	149 (0.3)	0.68	0.76
		CT	255 (0.52)	247 (0.5)		
		TT	100 (0.2)	97 (0.2)		
	rs2236857	T	644 (0.65)	610 (0.62)	0.10	2.7
		C	342 (0.35)	378 (0.38)		
		TT	218 (0.44)	188 (0.38)	0.14	3.9
		TC	208 (0.42)	234 (0.47)		
		CC	67 (0.14)	72 (0.15)		
	rs2236855	C	649 (0.66)	612 (0.62)	0.06	3.6
		A	333 (0.34)	376 (0.38)		
		CC	222 (0.45)	189 (0.38)	0.08	4.9
		CA	205 (0.42)	234 (0.47)		
		AA	64 (0.13)	71 (0.14)		
	rs2298896	T	573 (0.59)	539 (0.55)	0.05	3.6
		G	401 (0.41)	449 (0.45)		
		TT	174 (0.36)	150 (0.3)	0.16	3.6
		TG	225 (0.46)	239 (0.48)		
		GG	88 (0.18)	105 (0.21)		
	rs421300	A	559 (0.58)	540 (0.55)	0.18	1.8
		G	407 (0.42)	444 (0.45)		
		AA	165 (0.34)	154 (0.31)	0.40	1.8
		AG	229 (0.47)	232 (0.47)		
		GG	89 (0.18)	106 (0.22)		
	rs529520	A	523 (0.54)	556 (0.57)	0.19	1.7
		C	447 (0.46)	422 (0.43)		
		AA	147 (0.3)	168 (0.34)	0.39	1.8
		AC	229 (0.47)	220 (0.45)		
		CC	109 (0.22)	101 (0.21)		
	rs12749204	A	762 (0.77)	745 (0.75)	0.33	0.95
		G	226 (0.23)	245 (0.25)		
		AA	296 (0.6)	282 (0.57)	0.62	0.94
		AG	170 (0.34)	181 (0.37)		
		GG	28 (0.06)	32 (0.06)		
	rs2234918	C	536 (0.54)	536 (0.54)	0.96	0.002
		T	454 (0.46)	452 (0.46)		
		CC	146 (0.29)	147 (0.3)	0.99	0.01
		CT	244 (0.49)	242 (0.49)		
		TT	105 (0.21)	105 (0.21)		

*P-Value < 0.05 is considered as significant

*NA* Not Applicable

### Regression analysis

In this study, regression analysis was done to detect the relationship between four major features that were significant for drug addiction among cases and the selected SNPs. Tables 3, 4, and 5 depict the outcome of regression analysis. However, age of the addicts, smoking and marital status revealed a relationship with different genotypes of some candidate SNPs of the investigated genes was only significant for the risk of drug addiction among males Jordanians.

**Table 3:** Regression analysis of the association between drug addiction features and candidate SNPs of *OPRM1* gene

SNP ID	Covariate	Regression coefficients B B vs Expected. B*		***P***-value**
rs648893	Age	0.42	1.53	< 0.0001
	Smoking	−1.27	0.28	< 0.0001
	Work	0.10	1.10	0.55
	Marital status	−1.34	0.26	< 0.0001
	GG	−0.28	0.75	0.33
	AG	0.11	1.11	0.44
	AA	reference		
rs609148	Age	0.42	1.52	< 0.0001
	Smoking	−1.27	0.28	< 0.0001
	Work	0.08	1.09	0.61
	Marital status	−1.33	0.26	< 0.0001
	GG	0.30	1.35	0.27
	AG	0.29	1.33	0.31
	AA	reference		
rs3823010	Age	0.42	1.52	< 0.0001
	Smoking	−1.28	0.27	< 0.0001
	Work	0.10	1.11	0.53
	Marital status	−1.34	0.26	< 0.0001
	GG	0.07	1.07	0.92
	AG	0.06	1.06	0.93
	AA	reference		
rs1799971	Age	0.42	1.52	< 0.0001
	Smoking	−1.28	0.27	< 0.0001
	Work	0.11	1.12	0.50
	Marital status	−1.34	0.26	< 0.0001
	GG	0.73	2.08	0.23
	AG	0.47	1.61	0.01
	AA	reference		
rs524731	Age	0.42	1.53	< 0.0001
	Smoking	−1.30	0.27	< 0.0001
	Work	0.06	1.07	0.62
	Marital status	−1.35	0.25	< 0.0001
	CC	−0.04	0.95	0.90
	CA	−0.06	0.93	0.87
	AA	reference		
rs511435	Age	0.42	1.52	< 0.0001
	Smoking	−1.30	0.27	< 0.0001
	Work	0.08	1.08	0.69
	Marital status	− 1.33	0.26	< 0.0001
	CC	0.18	1.20	0.63
	CT	0.15	1.16	0.70
	TT	reference		
rs1381376	Age	0.41	1.51	< 0.0001
	Smoking	−1.27	0.28	< 0.0001
	Work	0.11	1.11	0.53
	Marital status	−1.32	0.26	< 0.0001
	CC	0.33	1.39	0.65
	CT	0.16	1.18	0.82
	TT	reference		
rs3778156	Age	0.44	1.55	< 0.0001
	Smoking	−1.26	0.28	< 0.0001
	Work	0.10	1.10	0.56
	Marital status	−1.37	0.25	< 0.0001
	GG	0.03	1.03	0.96
	AG	−0.19	0.82	0.31
	AA	reference		
rs2075572	Age	0.42	1.524	< 0.0001
	Smoking	−1.26	0.283	< 0.0001
	Work	0.10	1.112	0.53
	Marital status	−1.34	0.262	< 0.0001
	CC	−0.18	0.833	0.34
	CG	−0.11	0.896	0.50
	GG	reference		
0063rs548646	Age	0.43	1.54	< 0.0001
	Smoking	−1.29	0.27	< 0.0001
	Work	0.10	1.11	0.54
	Marital status	−1.31	0.26	< 0.0001
	CC	0.05	1.05	0.81
	CT	0.01	1.01	0.96
	TT	reference		
rs671531	Age	0.42	1.52	< 0.0001
	Smoking	−1.25	0.28	< 0.0001
	Work	0.09	1.09	0.58
	Marital status	−1.34	0.26	< 0.0001
	GG	0.15	1.16	0.49
	AG	0.14	1.15	0.52
	AA	reference		

* Beta coefficients

** *P*-Value < 0.05 is considered as significant

**Table 4:** Regression analysis of the association between drug addiction features and candidate SNPs of *OPRK1* gene

SNP ID	Covariate	Regression coefficients B B vs Expected. B*		*P*-value**
rs12675595	Age	0.30	1.35	0.01
	Smoking	−1.25	0.28	< 0.0001
	Work	0.18	1.19	0.28
	Marital status	−0.98	0.37	< 0.0001
	GG	0.41	1.51	0.53
	AG	0.36	1.44	0.59
	AA	reference		
rs1051660	Age	−0.30	.735	0.01
	Smoking	1.25	3.515	< 0.0001
	Work	−0.18	.828	0.26
	Marital status	0.97	2.656	< 0.0001
	CC	0.20	1.223	0.730
	CA	0.32	1.390	0.581
	AA	reference		
rs6985606	Age	−0.30	0.73	0.01
	Smoking	1.24	3.46	< 0.0001
	Work	−0.16	0.84	0.32
	Marital status	0.96	2.62	< 0.0001
	CC	0.03	1.03	0.86
	CT	−0.01	0.98	0.96
	TT	reference		
rs997917	Age	− 0.29	0.74	0.01
	Smoking	1.26	3.55	< 0.0001
	Work	−0.17	0.84	0.30
	Marital status	0.94	2.56	< 0.0001
	CC	−0.20	0.81	0.28
	CT	−0.25	0.77	0.10
	TT	reference		
rs702764	Age	−0.31	0.73	0.01
	Smoking	1.26	3.55	< 0.0001
	Work	−0.17	0.84	0.30
	Marital status	0.97	2.64	< 0.0001
	CC	−0.09	0.91	0.78
	CT	−0.15	0.86	0.31
	TT	reference		
rs963549	Age	−0.30	0.73	0.01
	Smoking	1.26	3.54	< 0.0001
	Work	−0.18	0.83	0.28
	Marital status	0.96	2.61	< 0.0001
	CC	0.01	1.01	0.97
	CT	−0.09	0.90	0.78
	TT	reference		

* Beta coefficients

** *P*-Value < 0.05 is considered as significant

**Table 5:** Regression analysis of the association between drug addiction features and candidate SNPs of *OPRD1* gene

SNP ID	Covariate	Regression coefficients B B vs Expected. B*		P-value**
rs569356	Age	0.42	1.52	< 0.0001
	Smoking	−1.25	0.28	< 0.0001
	Work	0.11	1.12	0.50
	Marital status	−1.34	0.26	< 0.0001
	GG	0.50	1.65	0.59
	AG	−0.05	0.95	0.80
	AA	reference		
rs1042114	Age	0.30	1.36	0.01
	Smoking	−1.28	0.27	< 0.0001
	Work	0.17	1.19	0.29
	Marital status	−0.96	0.38	< 0.0001
	GG	−0.27	0.75	0.72
	TG	0.01	1.01	0.95
	TT	reference		
rs2236857	Age	0.30	1.35	0.01
	Smoking	−1.25	0.28	< 0.0001
	Work	0.16	1.18	0.32
	Marital status	−0.95	0.38	< 0.0001
	CC	−0.22	0.80	0.80
	TC	−0.25	0.77	0.77
	TT	reference		
rs2236855	Age	0.31	1.36	0.01
	Smoking	−1.24	0.288	< 0.0001
	Work	0.17	1.19	0.29
	Marital status	−0.96	0.38	< 0.0001
	CC	0.27	1.31	1.31
	CA	−0.02	0.97	0.97
	AA	reference		
rs2298896	Age	0.30	1.35	0.01
	Smoking	−1.26	0.28	< 0.0001
	Work	0.18	1.20	0.27
	Marital status	− 0.95	0.38	< 0.0001
	GG	−0.33	0.71	0.08
	TG	−0.26	0.77	0.09
	TT	reference		
rs421300	Age	0.31	1.37	0.004
	Smoking	−1.24	0.28	< 0.0001
	Work	0.19	1.20	0.26
	Marital status	−0.97	0.37	< 0.0001
	GG	−0.25	0.77	0.19
	GA	−0.10	0.90	0.51
	AA	reference		
rs529520	Age	0.31	1.37	0.004
	Smoking	−1.23	0.29	< 0.0001
	Work	0.18	1.20	0.27
	Marital status	−0.94	0.38	< 0.0001
	CC	0.23	1.27	0.20
	CA	0.15	1.16	0.32
	AA	reference		
rs12749204	Age	0.29	1.34	0.01
	Smoking	−1.25	0.28	<0.0001
	Work	0.17	1.19	0.28
	Marital status	−0.96	0.38	<0.0001
	GG	− 0.25	0.77	0.37
	AG	−0.11	0.89	0.42
	AA	reference		
rs2234918	Age	0.31	1.36	0.004
	Smoking	−1.27	0.28	<0.0001
	Work	0.19	1.21	0.25
	Marital status	−0.97	0.37	<0.0001
	CC	−0.13	0.87	0.50
	CT	−0.18	0.83	0.30
	TT	reference		

* Beta coefficients

** *P*-Value < 0.05 is considered as significant

## Discussion

Drug addiction is a chronic disease that involves both the environmental and genetic factors [24]. Despite the vague knowledge about the robust role of the genetic parameters in drug addiction risk, several approaches were extensively investigated to put the baseline of genetic predisposition to drug addiction such as family, twin, and adoption studies [25–27]. This study focus on investigating the impact of many Single nucleotide variants within critical genes on drug addiction among Jordanians. Several previous studies conducted on Jordanian Arabs have investigated the involvement of candidate genes in drug addiction including *DRD4*, 5-HTTLPR [28] *SLC6A4* [29] and *OPRM1* [3, 30, 31]*.*

Our findings revealed that the *OPRM1* gene may be implicated in drug addiction among Jordanians, in particular the functioning SNP (rs1799971) found to be significantly associated with drug addiction which is in consistence with a Chinese study on336 Han heroin addicts [32]. In this study, the distribution of the variant genotype GG of rs1799971 within cases was twice it within controls which propose that the G allele of (rs1799971) within *OPRM1* gene may influence drug addiction increased risk. The aforementioned SNP is a missense variant located on exon 1 and encodes for a change from asparagine to aspartate amino acids. It has been suggested that this change results in removing an N-glycosylation site in the extracellular domain which alters the activity of endogenous opioid binding receptors [33]. These actions end in pain harbor and reduction in the response to analgesic drugs [9, 32] furthermore, it tend to increase administration of opioids [31]. In contrast to this study finding [11, 13] reported no evidence of an association between rs1799971 polymorphism and opioid addiction among African-Americans/ European-Americans (EA) and Japanese population.

rs648893 (C/T) polymorphism an Intronic SNP of the *OPRM1* gene that implicated in DNA splicing has been recently nominated as candidate for substance dependence including drugs [34]. In accordance to our study, [34, 35] found that the rs648893 of *OPRM1* was not associated with drug addiction among Iranian population and European Americans respectively. After all, ethnic variations reflect the differences in the drug addiction etiology among populations. Another significant outcome of this recent study, we found that one block of the *OPRM1* gene (AGGGCGACCCC) exhibits significant association with drug addiction among Jordanian males.

In this study, none of the investigated SNPs of both *OPRD1* and *OPRK1* genes were in correlation with drug addiction among Jordanians. However, in a review of several studies about genetic variants of addiction susceptibility summarizes that both rs1051660 and rs1051660 of *OPRK1* are involved in drug addiction [7], while rs1042114 polymorphism of *OPRD1* was significantly correlated with substance dependence disorder among EA [19].

On the other hand, our regression analysis declared that the relationship between the age of addicts, smoking and marital status with genetic variants within *OPRM1*, *OPRD1* and *OPRK1* genes may be implicated in drug addiction risk. Remarkably, it has been reported that suicide attempts can be induced by drug addiction disorder. In regard, no correlation between significant OPRM1 SNPs (rs1799971, rs609148 and rs648893) and suicide attempts behavior among 426 European-Americans addicts was found [36]. However, the real relation between genetic factors and clinical features of drug addiction is not fully elucidated, more studies and analyses are needed to understand the biological etiology of the disorder.

## Conclusion

In conclusion, we propose that rs1799971 of the *OPRM1* gene is a genetic risk factor for drug addiction among Jordanian males. To avoid genotyping bias in this study, we excluded the genetic association outcome caused by population stratification. This study was the first to investigate a genetic association of opioid gene variations with drug addiction in males of Arab descent. These findings may provide crucial knowledge to understand the drug addiction mechanisms in Middle Eastern population of Arab descent. Overall, the current study has examined three genes of opioid receptors that appear to be involved in the vulnerability to and the treatment of drug addiction in a Middle Eastern population of Arab descent. Finally, the results of this study provided additional clinical, epidemiological and genetic knowledge that may be useful in the context of further genetic and pharmacogenetic analyses to reduce the severity of drug consumption and improve drug abstinence.

## Supplementary Information

**Additional file 1: Table S1.** Different genetic models analysis between candidate gene SNPs and drug addiction. **TableS2.** Association between drug addiction and different haplotypes

## Data Availability

The complete processed SNP genotypic data for the three OPR genes is available as a supplementary file. (https://mega.nz/file/YMBnxIAK#3AiyPeLMzPhbsGHrdZnMUQId-y6sHOSx7-OXzkjHYkE).

## Abbreviations

OPR: Opioid receptors
HWE: Hardy-Weinberg equilibrium
DSM: Manual of Mental Disorders
DRC-PSD: Drug Rehabilitation Centre of the Jordanian Public Security Directorate
NCRA: National Centre for Rehabilitation of Addicts
