# Supplementary material for: Genetic susceptibility of opioid receptor genes polymorphism to drug addiction: A candidate-gene association study
Source: BMC Psychiatry. 2021 Jan 5;21:5. doi: 10.1186/s12888-020-03006-z (PMC7786995; doi:10.1186/s12888-020-03006-z)
Supplement: Supplementary file 1 — Additional file 1: Table S1. Different genetic models analysis between candidate gene SNPs and drug addiction. TableS2. Association between drug addiction and different haplotypes [file 12888_2020_3006_MOESM1_ESM.docx]

Genetic susceptibility of opioid receptor genes

polymorphism to drug addiction: A candidate-gene association study

Laith N. AL-Eitan^1,2^*, Doaa M. Rababa’h^1^, Mansour A Alghamdi^3,4^

*^1^ Department of Applied Biological Sciences, Jordan University of Science and Technology, Irbid 22110, Jordan*

*^2^ Department of Biotechnology and Genetic Engineering, Jordan University of Science and Technology, Irbid 22110, Jordan*

*^3^ Department of Anatomy, College of Medicine, King Khalid University, Abha, 61421, Saudi Arabia*

*^4^ Genomics and Personalized Medicine Unit, College of Medicine, King Khalid University, Abha, 61421, Saudi Arabia.*

***** Correspondence: Laith N. AL-Eitan, [lneitan@just.edu.jo](mailto:lneitan@just.edu.jo); Tel.: +962-2-7201000 ext.: 23464.

| **Table S1.** Different genetic models analysis between candidate gene SNPs and drug addiction. | | | | | | | | |
| --- | --- | --- | --- | --- | --- | --- | --- | --- |
| **Gene** | **SNP ID** | **Model** | **Genotype** | **Cases(498 %)** | **Controls 496%)** | **OR (95% CI)** | **P-Value** |  |
| ***OPRM1*** | rs648893 | Dominant | A/A  G/A-G/G | 267 (53.9%)  228 (46.1%) | 267 (53.9%)  228 (46.1%) | 1.00  1.00 (0.78-1.28) | 1.0 |  |
|  |  | Recessive | A/A-G/A  G/G | 467 (94.3%)  28 (5.7%) | 456 (92.1%)  39 (7.9%) | 1.00  0.70 (0.42-1.16) | 0.16 |  |
|  | rs609148 | Dominant | G/G  A/G-A/A | 267 (54.3%)  225 (45.7%) | 255 (51.6%)  239 (48.4%) | 1.00  0.90 (0.70-1.15) | 0.4 |  |
|  |  | Recessive | G/G-A/G  A/A | 462 (93.9%)  30 (6.1%) | 454 (91.9%)  40 (8.1%) | 1.00  0.74 (0.45-1.20) | 0.22 |  |
|  | rs3823010 | Dominant | G/G  G/A-A/A | 416 (84.5%)  76 (15.4%) | 411 (83%)  84 (17%) | 1.00  0.89 (0.64-1.25) | 0.52 |  |
|  |  | Recessive | G/G-G/AA/A | 489 (99.4%)  3 (0.6%) | 490 (99%)  5 (1%) | 1.00  0.60 (0.14-2.53) | 0.48 |  |
|  | rs1799971 | Dominant | A/A  G/A-G/G | 364 (73.7%)  130 (26.3%) | 404 (81.6%)  91 (18.4%) | 1.00  1.59 (1.17-2.15) | **0.003** |  |
|  |  | Recessive | A/A-G/A  G/G | 484 (98%)  10 (2%)) | 490 (99%)  5 (1%) | 1.00  2.02 (0.69-5.97) | 0.19 |  |
|  | rs511435 | Dominant | C/C  C/T-T/T | 353 (71.5%)  141 (28.5%) | 336 (68%)  158 (32%) | 1.00  0.85 (0.65-1.11) | 0.24 |  |
|  |  | Recessive | C/C-C/T  T/T | 480 (97.2%)  14 (2.8%) | 475 (96.2%)  19 (3.8%) | 1.00  0.73 (0.36-1.47) | 0.38 |  |
|  | rs524731 | Dominant | G/G  G/A-A/A | 230 (46.8%)  261 (53.2%) | 223 (45.1%)  271 (54.9%) | 1.00  0.93 (0.73-1.20) | 0.59 |  |
|  |  | Recessive | G/G-G/AA/A | 439 (89.4%)  52 (10.6%) | 430 (87%)  64 (13%) | 1.00  0.80 (0.54-1.17) | 0.25 |  |
|  | rs1381376 | Dominant | C/C  C/T-T/T | 418 (85%)  74 (15%) | 405 (81.8%)  90 (18.2%) | 1.00  0.80 (0.57-1.12) | 0.18 |  |
|  |  | Recessive | C/C-C/T  T/T | 489 (99.4%  3 (0.6%) | 488 (98.6%)  7 (1.4%) | 1.00  0.43 (0.11-1.66) | 0.2 |  |
|  | rs3778156 | Dominant | A/A  G/A-G/G | 419 (84.8%)  75 (15.2%) | 409 (82.6%)  86 (17.4%) | 1.00  0.85 (0.61-1.19) | 0.35 |  |
|  |  | Recessive | A/A-G/A  G/G | 490 (99.2%)  4 (0.8%) | 490 (99%)  5 (1%) | 1.00  0.80 (0.21-3.00) | 0.74 |  |
|  | rs2075572 | Dominant | C/C  C/G-G/G | 160 (32.5%)  332 (67.5%) | 142 (28.7%)  353 (71.3%) | 1.00  0.83 (0.64-1.09) | 0.19 |  |
|  |  | Recessive | C/C-C/G  G/G | 390 (79.3%)  102 (20.7%) | 376 (76%)  119 (24%) | 1.00  0.83 (0.61-1.12) | 0.21 |  |
|  | rs548646 | Dominant | C/C  C/T-T/T | 223 (46.6%)  256 (53.4%) | 215 (44.1%)  273 (55.9%) | 1.00  0.90 (0.70-1.16) | 0.44 |  |
|  |  | Recessive | C/C-C/T  T/T | 425 (88.7%)  54 (11.3%) | 425 (87.1%)  63 (12.9%) | 1.00  0.86 (0.58-1.26) | 0.44 |  |
|  | rs671531 | Dominant | G/G  G/A-A/A | 230 (46.8%)  261 (53.2%) | 223 (45.1%)  271 (54.9%) | 1.00  0.93 (0.73-1.20) | 0.59 |  |
|  |  | Recessive | G/G-G/A  A/A | 439 (89.4%)  52 (10.6%) | 430 (87%)  64 (13%) | 1.00  0.80 (0.54-1.17) | 0.25 |  |
| ***OPRK1*** | rs12675595 | Dominant | G/G  G/A-A/A | 406 (81.8%)  90 (18.1%) | 405 (81.8%)  90 (18.2%) | 1.00  1.00 (0.72-1.38) | 0.99 |  |
|  |  | Recessive | G/G-G/A  A/A | 491 (99%)  5 (1%) | 489 (98.8%)  6 (1.2%) | 1.00  0.83 (0.25-2.74) | 0.76 |  |
|  | rs1051660 | Dominant | C/C  C/A-A/A | 394 (79.6%)  101 (20.4%) | 383 (77.4%)  112 (22.6%) | 1.00  0.88 (0.65-1.19) | 0.39 |  |
|  |  | Recessive | C/C-C/A  A/A | 488 (98.6%)  7 (1.4%) | 486 (98.2%)  9 (1.8%) | 1.00  0.77 (0.29-2.10) | 0.61 |  |
|  | rs6985606 | Dominant | C/C  C/T-T/T | 217 (44.3%)  273 (55.7%) | 223 (45.1%)  271 (54.9%) | 1.00  1.04 (0.81-1.33) | 0.79 |  |
|  |  | Recessive | C/C-C/T  T/T | 436 (89%)  54 (11%) | 441 (89.3%)  53 (10.7%) | 1.00  1.03 (0.69-1.54) | 0.88 |  |
|  | rs997917 | Dominant | T/T  C/T-C/C | 139 (28.2%)  353 (71.8%) | 163 (33.1%)  329 (66.9%) | 1.00  1.26 (0.96-1.65) | 0.09 |  |
|  |  | Recessive | T/T-C/T  C/C | 390 (79.3%)  102 (20.7%) | 389 (79.1%)103 (20.9%) | 1.00  0.99 (0.73-1.34) | 0.94 |  |
|  | rs702764 | Dominant | T/T  C/T-C/C | 314 (63.4%)  181 (36.6%) | 320 (64.7%)  175 (35.4%) | 1.00  1.05 (0.81-1.37) | 0.69 |  |
|  |  | Recessive | T/T-C/T  C/C | 474 (95.8%)  21 (4.2%) | 470 (95%)  25 (5%) | 1.00  0.83 (0.46-1.51) | 0.55 |  |
|  | rs963549 | Dominant | C/C  C/T-T/T | 316 (64.2%)  176 (35.8%) | 316 (64.2%)  176 (35.8%) | 1.00  1.00 (0.77-1.30) | 1.0 |  |
|  |  | Recessive | C/C-C/T  T/T | 473 (96.1%)  19 (3.9%) | 467 (94.9%)  25 (5.1%) | 1.00  0.75 (0.41-1.38) | 0.35 |  |
| ***OPRD1*** | rs569356 | Dominant | A/A  G/A-G/G | 421 (85.6%)  71 (14.4%) | 423 (86.2%)  68 (13.8%) | 1.00  1.05 (0.73-1.50) | 0.79 |  |
|  |  | Recessive | A/A-G/A  G/G | 489 (99.4%)  3 (0.6%) | 489 (99.6%)  2 (0.4%) | 1.00  1.50 (0.25-9.02) | 0.65 |  |
|  | rs1042114 | Dominant | T/T  G/T-G/G | 421 (85.2%)  73 (14.8%) | 423 (85.6%)  71 (14.4%) | 1.00  1.03 (0.73-1.47) | 0.86 |  |
|  |  | Recessive | T/T-G/T  G/G | 491 (99.4%)  3 (0.6%) | 489 (99%)  5 (1%) | 1.00  0.60 (0.14-2.51) | 0.48 |  |
|  | rs678849 | Dominant | C/C  C/T-T/T | 136 (27.7%)  355 (72.3%) | 149 (30.2%)  344 (69.8%) | 1.00  1.13 (0.86-1.49) | 0.38 |  |
|  |  | Recessive | C/C-C/T  T/T | 391 (79.6%)  100 (20.4%) | 396 (80.3%)  97 (19.7%) | 1.00  1.04 (0.76-1.43) | 0.79 |  |
|  | rs2236857 | Dominant | T/T  C/T-C/C | 275 (55.8%)  426 (86.4%) | 188 (38.1%)  306 (61.9%) | 1.00  0.78 (0.60-1.00) | 0.05 |  |
|  |  | Recessive | T/T-C/T  C/C | 426 (86.4%)  67 (13.6%) | 422 (85.4%)  72 (14.6%) | 1.00  0.92 (0.64-1.32) | 0.66 |  |
|  | rs2236855 | Dominant | C/C  C/A-A/A | 222 (45.2%)  269 (54.8%) | 189 (38.3%)  305 (61.7%) | 1.00  0.75 (0.58-0.97) | 0.03 |  |
|  |  | Recessive | C/C-C/A  A/A | 427 (87%)  64 (13%) | 423 (85.6%)  71 (14.4%) | 1.00  0.89 (0.62-1.28) | 0.54 |  |
|  | rs2298896 | Dominant | T/T  G/T-G/G | 174 (35.7%)  313 (64.3%) | 150 (30.4%)  344 (69.6%) | 1.00  0.78 (0.60-1.02) | 0.07 |  |
|  |  | Recessive | T/T-G/T  G/G | 399 (81.9%)  88 (18.1%) | 389 (78.7%)  105 (21.3%) | 1.00  0.82 (0.60-1.12) | 0.21 |  |
|  | rs421300 | Dominant | A/A  G/A-G/G | 165 (34.2%)  318 (65.8%) | 154 (31.3%)  338 (68.7%) | 1.00  0.88 (0.67-1.15) | 0.34 |  |
|  |  | Recessive | A/A-G/A  G/G | 394 (81.6%)  89 (18.4%) | 386 (78.5%)  106 (21.5%) | 1.00  0.82 (0.60-1.13) | 0.22 |  |
|  | rs529520 | Dominant | A/A  C/A-C/C | 147 (30.3%)  338 (69.7%) | 168 (34.4%)  321 (65.6%) | 1.00  1.20 (0.92-1.57) | 0.18 |  |
|  |  | Recessive | A/A-C/A  C/C | 376 (77.5%)  109 (22.5%) | 388 (79.3%)  101 (20.6%) | 1.00  1.11 (0.82-1.51) | 0.49 |  |
|  | rs12749204 | Dominant | A/A  G/A-G/G | 296 (59.9%)  198 (40.1%) | 282 (57%)  213 (43%) | 1.00  0.89 (0.69-1.14) | 0.35 |  |
|  |  | Recessive | A/A-G/A  G/G | 466 (94.3%)  28 (5.7%) | 463 (93.5%)  32 (6.5%) | 1.00  0.87 (0.52-1.47) | 0.6 |  |
|  | rs2234918 | Dominant | C/C  C/T-T/T | 146 (29.5%)  349 (70.5%) | 147 (29.8%)  347 (70.2%) | 1.00  1.01 (0.77-1.33) | 0.93 |  |
|  |  | Recessive | C/C-C/T  T/T | 390 (78.8%)  105 (21.2%) | 252 (51%)  242 (49%) | 1.00  1.00 (0.74-1.35) | 0.99 |  |
| *P*- Value <0.025 was considered as significant after performing Bonferroni correction.  OD: odd ratio.  CI: Confidence interval. | | | | | | | | |

| **TableS2.** Association between drug addiction and different haplotypes. | | | | |
| --- | --- | --- | --- | --- |
| **Haplotype blocks** | **Frequency**  **of block** | **Frequency ratio % (case: control)** | **Odds ratio**  **(95%) CI** | ***P*-value** |
| ***OPRM1 (*Global haplotype association p- value:0.14)** | | | | |
| AGGGCAACCCC | 0.3647 | 0.3496:0.3771 | 1 | NA |
| GAGACAAGCT | 0.24 | 0.2355: 0.2472 | 1.01 (0.80 - 1.28) | 0.94 |
| AGGGCGACCCC | 0.1134 | 0.1354:0.0938 | 1.56 (1.15 - 2.12) | **0.01** |
| AGGGTAAGCCA | 0.0671 | 0.0652:0.069 | 0.99 (0.68 - 1.42) | 0.94 |
| AGGGCAAGCCC | 0.0508 | 0.0521:0.0494 | 1.08 (0.70 - 1.65) | 0.73 |
| AGAGTAGCTCA | 0.0347 | 0.0345: 0.0339 | 1.00 (0.60 - 1.67) | 1.00 |
| AGAATAGGTTA | 0.031 | 0.036: 0.031 | 0.78 (0.46 - 1.33) | 0.36 |
| ***OPRD1 (*Global haplotype association p- value:0.48)** | | | | |
| TTAACTTCA | 0.1951 | 0.1938:0.1986 | 1 | NA |
| TTAACTTCCA | 0.1752 | 0.1883:0.1603 | 1.18 (0.83 - 1.68) | 0.36 |
| TGGGACATA | 0.1191 | 0.1156:0.125 | 0.92 (0.63 - 1.36) | 0.69 |
| TGGAACCACA | 0.0976 | 0.0837:0.113 | 0.77 (0.53 - 1.12) | 0.18 |
| TGGGACCACA | 0.0885 | 0.0915:0.0836 | 1.14 (0.77 - 1.69) | 0.51 |
| GTAAACTCCG | 0.0492 | 0.0459:0.0518 | 0.89 (0.55 - 1.46) | 0.66 |
| TGGAACCATA | 0.0329 | 0.0285:0.0354 | 0.81 (0.40 - 1.67) | 0.58 |
| TTAAACTCCA | 0.0312 | 0.0326:0.0296 | 1.20 (0.67 - 2.15) | 0.55 |
| TTAACCTCTA | 0.0293 | 0.0314:0.0272 | 1.22 (0.67 - 2.23) | 0.51 |
| GTAAACTCTG | 0.024 | 0.0286:0.0196 | 1.55 (0.75 - 3.19) | 0.23 |
| TGGAATTCCA | 0.0186 | 0.0154:0.0215 | 0.80 (0.35 - 1.85) | 0.60 |
| TGGAATTCTA | 0.0185 | 0.0264:0.0116 | 2.02 (0.85 - 4.81) | 0.11 |
| TTAACCTCCA | 0.0173 | 0.0189:0.0157 | 1.08 (0.48 - 2.47) | 0.85 |
| TGGGATTCCA | 0.0167 | 0.0114:0.0209 | 0.63 (0.28 - 1.42) | 0.27 |
| ***OPRK1 (*Global haplotype association p- value: 0.75)** | | | | |
| CTTTGC | 0.3255 | 0.3287:0.3227 | 1 | NA |
| CCTTGC | 0.2164 | 0.2042:0.2287 | 0.88 (0.70 - 1.12) | 0.3 |
| CCCCGC | 0.1356 | 0.1405:0.1293 | 1.06 (0.80 - 1.40) | 0.68 |
| CCCTGC | 0.1034 | 0.1151:0.0922 | 1.20 (0.87 - 1.65) | 0.27 |
| CCCTAC | 0.0827 | 0.0836:0.0822 | 1.00 (0.70 - 1.44) | 0.98 |
| ACCTGC | 0.0628 | 0.0604:0.065 | 0.93 (0.62 - 1.38) | 0.71 |
| ACCCGT | 0.0499 | 0.0462:0.0537 | 0.84 (0.54 - 1.32) | 0.45 |
| CCCCAT | 0.0122 | 0.0108:0.0137 | 0.75 (0.31 - 1.86) | 0.54 |
